# Prevalence, Antimicrobial Susceptibility, and Hygienic Practices of *Escherichia coli* O157:H7 in Raw Meat, Bahir Dar City, Ethiopia

**DOI:** 10.1002/vms3.71246

**Published:** 2026-09-24

**Authors:** Wogayehu Asefa, Habtamu Tassew, Tiliksew Bialfew, Esayas Gelaye, Bizuneh Tsehayneh Azene

**Affiliations:** ^1^ Amhara Livestock and Fisheries Resources Development Bureau Bahir Dar Ethiopia; ^2^ School of Veterinary Medicine Bahir Dar University Bahir Dar Ethiopia; ^3^ National Veterinary Institute Federal Democratic Republic of Ethiopia Addis Ababa Ethiopia

**Keywords:** antimicrobial susceptibility, butcher houses, *Escherichia coli* O157:H7, prevalence, raw meat

## Abstract

**Introduction:**

Escherichia coli O157: H7 is a globally important zoonotic pathogen responsible for the majority of severe cases of human enterohemorrhagic *Escherichia coli* (EHEC) disease. The transmission of this pathogen is exacerbated in Ethiopia due to the common habit of consuming raw and/or undercooked meat.

**Objective:**

This study was conducted to isolate *E. coli* O157:H7, detect virulence genes, and assess the antimicrobial susceptibility patterns of isolates recovered from ready‐to‐eat raw meat (*kurt*) sold in Bahir Dar City, Ethiopia.

**Methodology:**

A cross‐sectional study design with a simple random sampling technique was employed to collect questionnaire data and raw meat samples from butcher houses. A total of 74 meat samples were collected and processed using standard microbiological methods, and genomic DNA from positive isolates was analyzed by polymerase chain reaction (PCR). All data collected during the study period were coded and entered into an Excel spreadsheet and analyzed using STATA version 12.

**Results:**

Of the 74 examined samples, *E. coli* O157:H7 was detected in 21 (28.4%). Out of 21 isolates, 10 (47.6%) carried at least one of the investigated virulence genes, with detection rates of 100% for the *stx1* and 70% for the *eaeA* genes. Only 16.2% of meat handlers had received training on safe food handling. Lack of training was associated with higher odds of *E. coli* O157:H7 contamination (AOR = 0.16, *p* = 0.028). The presence of *E. coli* O157:H7 was also significantly associated with the practice of workers collecting money from the customers while cutting meat (AOR [95% CI] = 3.7 [1.01–11.48]). Antibiotic profiling showed that the resistance rate ranged from 4.8% to 33.3%. About six (28.6%) isolates were resistant to two antimicrobial agents, amoxicillin and chloramphenicol.

**Conclusion:**

The detection of *E. coli* O157:H7 in nearly one‐third of the samples and the occurrence of virulence genes in half of the isolates indicate the potential risk of foodborne infection in the study area. Therefore, food safety awareness and training for meat handlers should be encouraged to reduce the spread of this pathogen in Bahir Dar City.

## Graphical abstract

*Escherichia coli* O157:H7 was detected in 28.4% of raw meat samples from Bahir Dar City, with 47.6% of isolates carrying at least one *stx1 or eaeA* virulence gene. Poor hygiene practices and antimicrobial resistance highlight the need for strengthened meat safety surveillance and intervention.

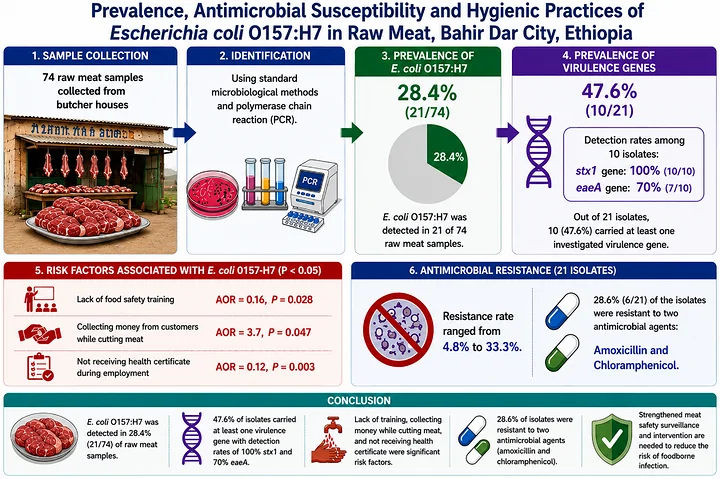

## 1 Introduction

Foodborne pathogens are the leading causes of illness and death in developing countries, costing billions of dollars in medical care and social costs (Ali et al. 2010). The consumption of raw or undercooked contaminated meat is one of the most common means of transmission of potentially pathogenic zoonotic bacteria, primarily *E* *scherichia* *coli*, Salmonella, and Campylobacter species (Humphrey and Jørgensen 2006; Kim et al. 2019).

Among the foodborne bacteria, *E. coli* is an important pathogen that can cause diarrhoea, haemorrhagic colitis, and haemolytic uraemic syndrome in humans (Zhang et al. 2015). *Escherichia coli* O157:H7 is the predominant serotype causing severe human infections that have low infectious doses and are transmitted through different mechanisms, including food and water (Croxen et al. 2013). The pathogenicity of *E. coli* O157:H7 is associated with virulence factors of Shiga toxins 1 and 2 (encoded by *stx1* and *stx2* genes), intimin (encoded by *eaeA* gene), and enterohaemolysin (encoded by *hlyA* genes) (Zhang et al. 2015).

The development of antibiotic resistance among bacteria, such as *E. coli*, poses a significant public health concern. Moreover, the effectiveness of treatments and the ability to control infectious diseases in both animals and humans are severely hampered due to the rapid development of multidrug resistance (Thaker et al. 2012).

Ethiopia is located in sub‐Saharan Africa, a region with a high burden of foodborne diseases, with *E. coli* being one of the leading causes of foodborne illnesses, and disability adjusted life years (Havelaar et al. 2015). The transmission of foodborne pathogens, including *E. coli* O157:H7, is exacerbated in Ethiopia due to the common habit of consuming raw and/or undercooked meat in the form of “*kitfo*” (minced raw meat mixed with a chili powder‐based spice blend and a clarified butter infused with herbs and spices) and “*kurt*” (raw sliced meat consumed with hot pepper and mustard) (Bekele et al. 2014).

Despite the widespread presence of the problem, only a few studies have been conducted on *E. coli* O157: H7 in retail meat, particularly in Bahir Dar City where the consumption of raw beef is common. Moreover, previous studies rarely combined bacteriological isolation, virulence gene detection, antimicrobial susceptibility testing, and assessment of meat handling practices in the same investigation. Data generated in this study provide baseline information for Bahir Dar City and identify potential food safety gaps that can support local interventions. The aim of this study was to investigate the burden of *E. coli* O157:H7, examine virulence genes, and evaluate the antibiotic resistance of the isolates in ready‐to‐eat raw meat collected from butcher houses in Bahir Dar City.

## 2 Materials and Methods

### 2.1 Description of the Study Area

**FIGURE 1:**
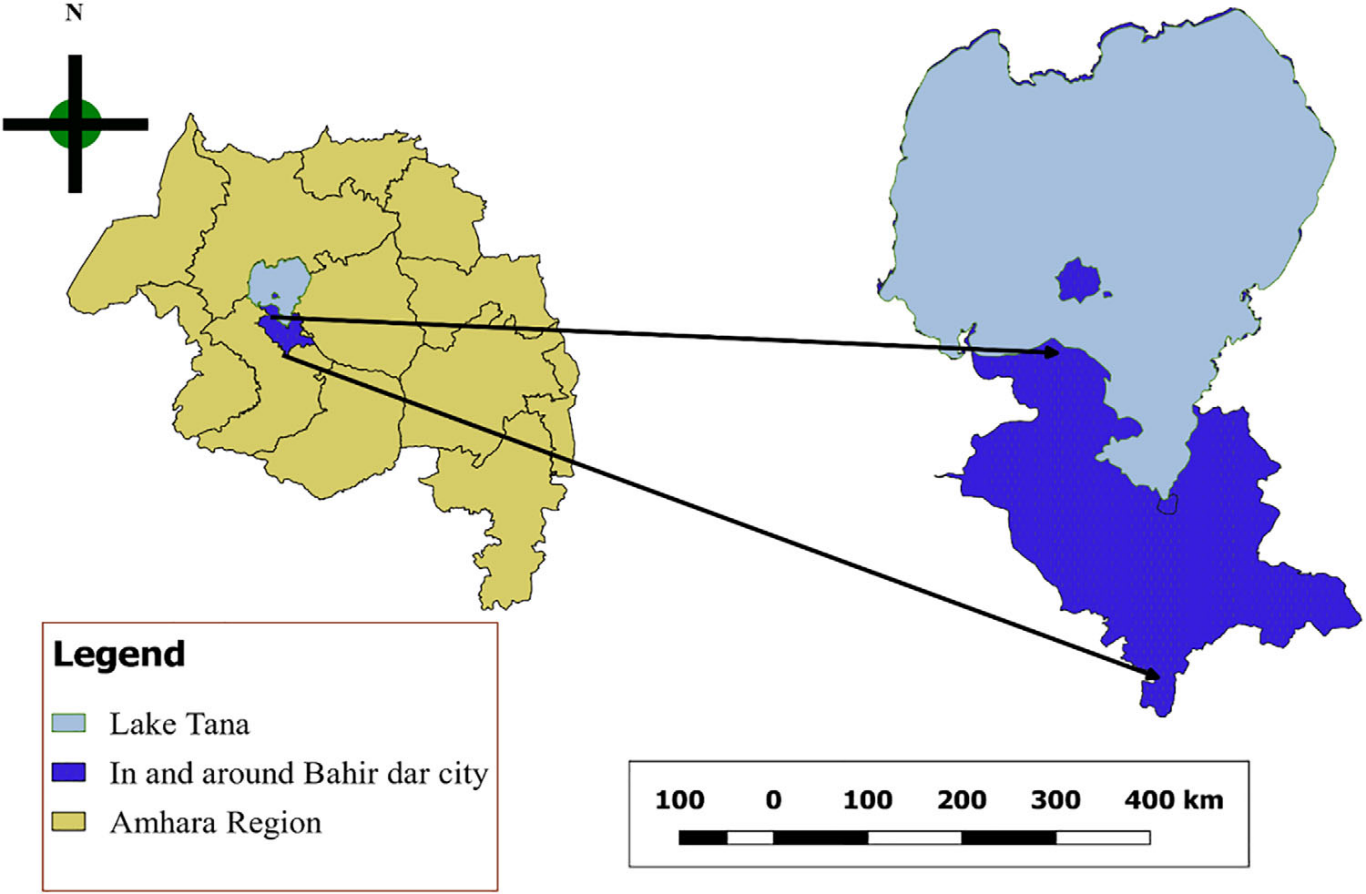
Map of the study area (drawn using QGIS 2.18.0).

The study was conducted in Bahir Dar City, which is the capital city of the Amhara National Regional State (ANRS), Ethiopia. Bahir Dar City is located 565 km from Addis Ababa, near Lake Tana and the headwaters of the Blue Nile, and is a major tourist destination (Figure 1). It is situated on a relatively flat plateau at approximately 11°36″ N latitude and 37°23″ E longitude. The altitude of this city ranges from 1810 to 1850 m above sea level, and the temperature ranges from 10 to 38°C.

### 2.2 Description of Butcher Houses

Butcher houses in Ethiopia are commonly small, privately owned establishments located in residential areas, local markets, or along busy streets to ensure easy access for consumers. They retail meat either by offering on‐site consumption services (commonly selling raw and cooked meat products such as *Kurt*, *Kitfo*, *Dulet*, *Leb leb*, *Tibs*) or by selling meat directly to consumers for home consumption. In butcher houses, carcasses are displayed openly at ambient temperature for visibility to customers and passers‐by, and meat cutting is performed manually using knives and wooden or plastic chopping blocks. Butcher houses are regulated by local municipal authorities and public health offices. In Bahir Dar City, there is one municipal abattoir and about 91 licensed butcher houses.

### 2.3 Study Design

A cross‐sectional study was conducted from November 2019 to March 2020 in Bahir Dar City to identify *E. coli* O157:H7, evaluate its antimicrobial susceptibility, and assess risk factors associated with contamination.

### 2.4 Sample Size Determination

The study targets butcher houses operating in Bahir Dar City, and the sample size for both the questionnaire and raw meat was determined by Yamane's sample size calculation formula, as indicated below (Yamane 1973).

(1) $$n=\frac{N}{1+N\left(e^{2}\right)}$$

where *n* is the sample size, N is the population size, and *e* is the margin of error.

Based on the above information, 74 butcher houses were included from a total of 91 licensed butcher houses operating in meat and meat products in Bahir Dar City.

### 2.5 Sampling Methods and Sample Collection

Butcher houses were selected using a simple random sampling technique, with their lists obtained from the Bahir Dar City Administration Municipal Office. To assess the butcher house workers' hygienic practices, pre‐tested structured questionnaires were administered through interviews, and direct observations were also conducted by preparing observation checklists.

Ready‐to‐eat raw meat samples chopped with a knife were collected and placed in sterile plastic bags. The samples were then transported in ice boxes to the Amhara Public Health Institute Laboratory for further analysis.

### 2.6 Isolation and Identification of *Escherichia coli* O157:H7

Isolation and identification of *E. coli* O157:H7 were performed according to the previously described methods, with minor modifications (International Organization for Standardization 2001; Karmi 2019; Mizuochi et al. 2016). From the collected samples, 25 g of minced meat was mixed with 225 mL of buffered peptone water in a sterile plastic bag and homogenized using a Stomacher 400 homogenizer for 2 min. A loopful of the sample was placed on the surface of Compact Dry EC medium to identify *E. coli*. Bacteria other than coliforms were inhibited, and those that resisted inhibition did not form the typical blue/blue‐purple or red/pink colonies. *Escherichia coli* formed blue/blue‐purple colonies, while other coliform bacteria formed red/pink colonies. Presumptive colonies on the Compact Dry EC medium were sub‐cultured onto Sorbitol MacConkey agar plates (CM0813; Oxoid Basingstoke). Non‐sorbitol fermenting colonies (pale colonies) presumptively identified as *E. coli O157* were transferred to nutrient agar (Oxoid, England) for further molecular characterization. Morphological characteristics as well as cultural and biochemical characteristics (fermentation of lactose and glucose using triple sugar iron agar, hydrogen sulphide [H_2_S] negative, production of indole [positive], methyl red test [positive], Voges‐Proskauer test [negative], and Simon citrate agar test [negative]) were assessed during laboratory testing (Quinn et al. 2011).

Virulence gene detection was conducted only for *stx1* and *eaeA* genes because primers for other genes were unavailable. DNA was extracted from all presumptive isolates following previously established protocols, with slight modifications (Abreham et al. 2019; Mazaheri et al. 2014). DNA was extracted from presumptive isolates using the boiling method. Pure colonies were homogenized in 100 µL of sterile distilled water in Eppendorf tubes. The resulting suspension was placed in a boiling water bath at 100°C for 10 min, and then centrifuged at 15,000 rpm for 5 minutes in a microcentrifuge (Peqlab). The supernatant containing bacterial DNA was transferred to a new tube and used for PCR detection of the *stx1* and *eaeA* genes.

PCR amplification was performed in an Eppendorf thermal cycler. The temperature conditions included an initial denaturation step at 95°C for 3 min, followed by a final extension step at 60°C for 5 min. Detailed primer sequences and PCR conditions are provided in the table 1.

**TABLE 1:** PCR primer sequences, conditions, and products used for the detection of virulence genes.

Target gene	Primer sequence	PCR conditions for 35 cycles									PCR product size (bp)	References
		Denaturation			Annealing			Extension				
		Temperature (°C)	Time (s)		Temperature (°C)		Time	Temperature (°C)	Time (s)			
*Stx1*	F‐CAACACTGGATGATCTCAG	95	40		55		30	72	60		349	Tassew (2015)
	R‐CCCCCTCAACTGCTAATA											
*eaeA*	F‐GTGGCGAATACTGGCGAGACT	96°C		10	50	5		60		60	890	Fagan et al. (1999)
	R‐CCCCATTCTTTTTCACCGTCG											

Abbreviations: F, forward primer; R, reverse primer.

The PCR products were separated by gel electrophoresis in a horizontal electrophoresis system on a 1.5% (w/v) agarose gel containing 3 µL of ethidium bromide for 55 min at 110V using 1X TAE buffer. Amplicon sizes were estimated by comparing them with a 100‐bp DNA molecular weight marker. Detection of the amplified DNA was conducted using a UV transilluminator (Unico, China). Photographs were taken using gel documentation system attached to a computer.

### 2.7 Antimicrobial Susceptibility Testing

Antimicrobial susceptibility testing was performed using kanamycin, amoxicillin, ciprofloxacin, gentamicin, chloramphenicol, and ceftriaxone using Mueller‐Hinton agar (MHA). These antibiotics were selected because they represented commonly prescribed antimicrobials from different antimicrobial classes in Ethiopian veterinary and human medicine during the study period and were available in the laboratory. Morphologically identical four to six bacterial colonies from overnight culture were transferred into tryptone soya broth and incubated at 37°C for 6 h. The turbidity of the culture broth was adjusted by comparing with 0.5 McFarland standards (approximately 3 × 10^8^ CFU/mL). A sterile cotton swab was immersed into the suspension and rotated against the side of the tube to remove the excess fluid and then swabbed in three directions uniformly on the surface of Mueller‐Hinton agar (Oxoid, UK) plates. After the plates dried, antimicrobial disks were gently pressed onto the agar to ensure firm contact with the agar surface and incubated at 37°C for 24 h. The zones of inhibition were measured with a caliper in millimetres and interpreted according to the Clinical and Laboratory Standards Institute (CLSI) as susceptible, intermediate, or resistant (CLSI 2019).

### 2.8 Data Quality Assurance

The quality of data and test reliability were ensured by following standard operating procedures and maintaining aseptic conditions through appropriate sterilization, flaming, and refrigeration procedures. Contamination checks were performed by incubating 5% of the batch media at 37°C for 18–24 h, and the performance of the media was checked using a standard *E. coli* strain (ATCC 25922). The PCR was performed for each virulence gene in a total reaction volume of 25 µL containing 5 µL of extracted DNA, 10 µL of PCR master mix, 1 µL of each primer, and nuclease‐free water. Sterile nuclease‐free water and a known *E. coli *O157:H7 isolate were used as negative and positive controls, respectively.

### 2.9 Data Analysis

All data collected during the study period were coded and entered into an excel spreadsheet, and analyzed using STATA version 12. The PCR product sizes were determined using the 100‐base pair marker.

## 3 Results

### 3.1 Participant Demographics

Among 74 interviewed butcher house workers, 76% were aged between 18 and 30 years, and the remaining participants were older than 30 years. On the other hand, from the total respondents, 72 (97.2%) were male. Three (4%) of respondents had no formal education, 42 (56.75%) had attended only primary school (up to grade 8), 22 (29.72%) had attended senior secondary school (from grade 9 to 12), and 7 (9.4%) had a diploma certificate and above education. Regarding work experience, 60% of the respondents had more than 3 years of experience.

### 3.2 Detection of *E. coli* O157:H7

**FIGURE 2:**
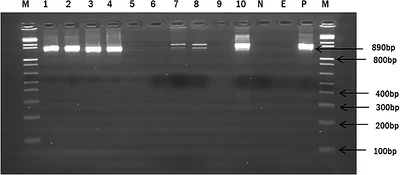
Representative gel image for agarose gel electrophoresis of amplified products of *eaeA* gene (890 bp) of *E. coli* O157:H7 isolates. Key: lane M: molecular ladder started 100 bp; lane N: negative control; lane P: positive control; lane E: extraction control; lanes 1–4, 7–8 and 10: positive samples for the *eaeA* gene.

**FIGURE 3:**
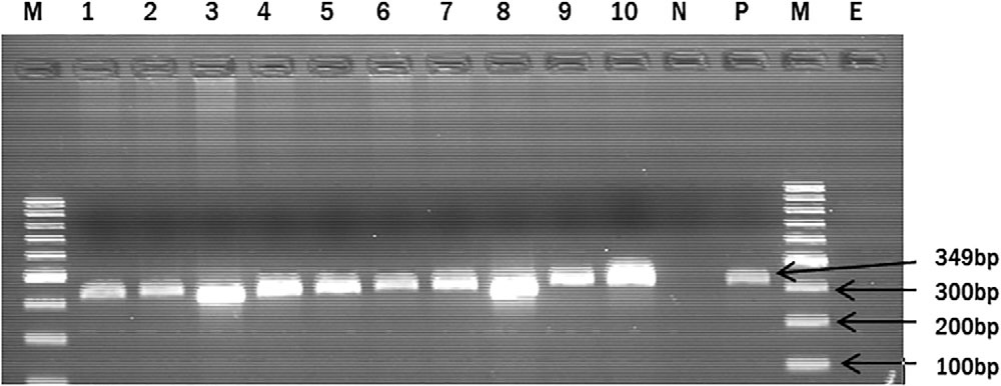
Representative gel image for agarose gel electrophoresis of amplified products of the *stx*_1_ gene (349 bp) of *E. coli* O157:H7 isolates. Key: lane M: molecular ladder started 100 bp; lane N: negative control; lane P: positive control; lane E: extraction control; lanes 1–10: positive samples for *stx1* gene.

Out of 74 samples, 21 (28.4%) were identified as presumptive *E. coli* O157:H7 based on culture and biochemical characteristics. Molecular analysis for virulence genes was conducted on all of the 21 isolates, and 10 (47.6%) isolates carried at least one of the investigated virulence genes (*stx1* and/or *eaeA*). Specifically, three isolates carried only *stx_1_* gene, and seven isolates carried both *stx_1_* and *eaeA* genes (Figures 2 and 3). The absence of these genes in the remaining isolates does not necessarily indicate the absence of pathogenic potential because only two virulence markers were investigated. Other important virulence genes including *stx2* and *hlyA* were not assessed.

### 3.3 Factors Associated With the Occurrence of *E. coli* in Raw Meat

Logistic regression analysis was performed to assess the association between potential risk factors and *E. coli* O157:H7 occurrence. Predictor variables with *p* < 0.25 in the univariable analysis were considered for inclusion in the multivariable logistic regression model. In this study, 81.1% of the meat handlers had undergone health‐status checks during employment; however, only 16.2% of the workers had received training about safe food handling and butchers who had received food‐safety training had 84% lower odds of *E. coli* O157:H7 contamination than butchers who had not received training (AOR = 0.16, *p* = 0.028). At the time of the butcher house visit, about 64.9% of the workers were collecting money from the customers. Workers who collected money while cutting meat had 3.7 times higher odds of contamination than those who used a separate money collector (cashier) after adjusting for other variables constant (AOR = 3.7, *p*‐value = 0.047) as indicated in Table 2.

**TABLE 2:** Univariate and multivariable logistic regression analysis of *E. coli* O157:H7 occurrence with meat handling practices of butchers in Bahir Dar City (*n* = 74).

Predictor variable	Frequency (%)	COR (95% CI)	P‐value	AOR (95% CI)	P‐value
Received a health certificate during employment	Yes: 60 (81.1)	0.09 (0.02–0.34)	0.00	0.12 (0.03–0.46)	0.003
Collecting money from customers while cutting meat	Yes: 48 (64.9)	4.11 (1.42–9.92)	0.01	3.7 (1.01–11.48)	0.047
Removing rings and watch while processing meat	Yes: 19 (25.7)	1.6 (0.48–5.81)	0.41	—	—
Removing work equipment when using toilets	Yes: 14 (18.9)	1.57 (0.39–6.31)	0.52	—	—
Washing gown after each day of work	Yes: 29 (39.2)	0.81 (0.29–2.26)	0.68	—	—
Received training on safe food handling	Yes: 12 (16.2)	0.21 (0.06–0.76)	0.017	0.16 (0.03–0.82)	0.028
Use of head covers and masks	Yes: 24 (32.4)	0.38 (0.14–1.13)	0.08	0.74 (0.19–2.88)	0.063

Abbreviations: AOR, adjusted odds ratio; COR, crude odds ratio.

### 3.4 Antimicrobial Susceptibility Profiling

*Escherichia coli* O157:H7 isolates were subjected to in vitro antimicrobial susceptibility testing to commonly used antibiotics. All isolates were susceptible to gentamicin, ciprofloxacin, and kanamycin. About 86% of the isolates were susceptible to ceftriaxone followed by chloramphenicol (81%) (Table 3). Six isolates were resistant to two drugs, and none of the isolates showed resistance against more than two drugs.

**TABLE 3:** Antibiotic profile of *E. coli* O157:H7 to selected antibiotics (Number of isolates N = 21).

No.	Antibiotic agent	Susceptibility	Intermediate	Resistant
1	Amoxicillin	13 (62%)	1 (4.8%)	7 (33.3%)
2	Ciprofloxacin	21 (100%)	—	—
3	Gentamicin	21 (100%)	—	—
4	Chloramphenicol	17 (81%)	—	6 (28.6%)
5	Ceftriaxone	18 (86%)	2 (9.5%)	1 (4.8%)
6	Kanamycin	21 (100%)	—	—

## 4 Discussion

Hygienic practices are a major concern among butcher‐house workers. However, hygienic practices were inadequate, as 83.8% of the respondents had not received training on food hygiene, safe meat handling, and personal hygiene in Bahir Dar City. This finding highlights inadequate implementation of food‐safety training, despite the recognized importance of training food handlers in food safety and hygiene practices that play an important role in reducing and preventing food poisoning through the production and distribution of safe food (Lazarević et al. 2013). Ansari‐Lari et al. (2010) also stated that knowledge of proper meat handling, handwashing, and other hygienic procedures by the meat handlers is very important since meat‐handlers can serve as vehicles for cross‐contamination and spread of foodborne pathogens.

In this study, about 64.9% of the butchers were collecting money while serving meat, which was higher than the previous report (47.3%), and 74.3% wore jewellery (i.e., rings, watches) which agreed with the study conducted in Mekele city that 78.9% of workers were wearing jewellery (Haileselassie et al. 2013). The practice of collecting money might have a significant contribution to the contamination of *E. coli* in this research. This finding is supported by the notion that money could be a prime transmission medium for various microorganisms and that it may pose a major health hazard, particularly when meat and money are handled together without washing hands (Girma et al. 2014). Only 32.4% of the butchers were using head covers and mouth masks. These poor practices of wearing protective clothing in the study area are inconsistent with the recommendations of the joint Food and Agriculture Organization of the United Nations/World Health Organization (FAO/WHO) Codex Alimentarius Commission and World Health Organization that contamination of meat and meat handlers could be prevented by wearing protective clothing, and the Ethiopian Ministry of Agriculture also recommends that protective clothes (such as gowns and hair covers) should be worn at all times when handling meat (FAO/WHO 2003; Ministry of Agriculture 2010).

The availability of safe food is a basic human right and guarantees the health of people. Safe food is a prerequisite for health, productivity, and development. In this study, the prevalence of *E. coli* O157:H7 was 28.38%, which is higher than that in previous studies conducted in Ethiopian retail houses, including 4.2% reported in Modjo and Bishftu (Hiko et al. 2008), 14.6% in Addis Ababa (Bekele et al. 2014), 2% in Hawassa (Atnafie et al. 2017), 4.5% in central Ethiopia (Beyi et al. 2017), and 4.5% in Jimma town (Sebsibe and Asfaw 2020). The reasons for this high prevalence might be due to poor hygienic practices. This interpretation is supported by the previous report that the high rate of meat contamination with *E. coli* may reflect inadequate hygienic and sanitary practices throughout the production chain, from slaughtering and transportation to butcher‐house handling and processing (Haileselassie et al. 2013). However, the finding of this study was much lower than the prevalence reports of 53% in Nigeria (Dahiru et al. 2010), 52.4% in Bangladesh (Islam et al. 2010), and 36.1% in Argentina (Llorente et al. 2014). The observed variation in the prevalence of *E. coli* O157:H7 in butcher houses could be due to the status of hygiene and sanitation practices of the butcher houses, the use of different methods of detection of isolates, differences in sample size, and the type of sample and how and when it was collected. For instance, the use of immuno‐magnetic separation may improve the sensitivity of detection, which was not used in the present study.

In the present study, 10 *E. coli* O157:H7 isolates carried virulence genes, and comparatively, the detection of the *stx_1_* gene was higher than *eaeA*. The detection rate of *stx1* (100%) in this study was higher than the reports in Saudi Arabia (45.45%) (Hessain et al. 2015). Relatively lower detection rates were reported for *stx_1_* (83.64%) and the *eaeA* gene (7.27%) from beef in India (Sethulekshmi et al. 2018). This finding indicates the potential of raw meat as a source of *E. coli* O157:H7 for human infections as a public health concern in the study area.

Antimicrobial resistance emerges from the use of antimicrobials in animals and humans and the subsequent transfer of resistance genes and bacteria among animals, humans, animal products, and the environment (Scott et al. 2002). In this research, the susceptibility rates ranged from 62% to 100%, whereas resistance rates ranged from 4.8% to 33.3%. The tested isolates showed the highest susceptibility (100%) for gentamicin, kanamycin, and ciprofloxacin. This finding was consistent with the previous reports conducted in Ethiopia (Hiko et al. 2008; Tassew 2015). On the other hand, the highest resistance was observed in amoxicillin (33.3%), followed by chloramphenicol (28.6%). This might be due to inappropriate or excessive use of antibiotics for therapeutic and prophylactic purposes forboth *E. coli* and other bacterial infections.

### 4.1 Limitations

The main limitations of the current study were limited resources for confirming the isolates by latex agglutination, serotyping, or sequencing and the unavailability of primers and reagents for detecting additional virulence and antibiotic resistance genes of *E. coli* O157:H7. Although samples were collected during 2019–2020, these findings provide valuable baseline data of *E. coli* O157:H7 and would be an input for food safety interventions.

## 5 Conclusion and Recommendations

In this study, *E. coli* O157:H7 was detected in nearly one‐third of the samples, and approximately half of the isolates carried virulence genes. The majority of food‐handling practices in the butcher houses could contribute to meat contamination. The detection of virulence genes indicates a potential public health concern and supports the need for continued surveillance and improved hygiene practices. Therefore, food safety training should be provided to butcher house owners and workers. Further studies should investigate additional virulence and antimicrobial resistance genes of *E. coli* O157:H7 isolates.

## Author Contributions

W.A. conceived the study, study design, resources, execution, data acquisition, analysis, and interpretation. H.T. was involved in data analysis, editing, and supervision. T.B. contributed to data analysis, editing, and supervision. E.G. was involved in data validation, resources, writing, and supervision. B.T.A. contributed to study design, execution, data analysis, and interpretation.

## Funding

This research did not receive any specific grant from funding agencies in the public, commercial, or not‐for‐profit sectors. The authors have nothing to report.

## Disclosure

This article has been published as a preprint (Asefa et al. 2025).

## Ethics Statement

The study was conducted after the proposal was ethically reviewed and approved by the Institutional Review Board of College of Agriculture and Environmental Sciences (code: CAES‐113/2019/1‐1‐3). A letter of support from Bahir Dar University (Ref No:‐ 1/974/1‐1‐3) was written to APHI. Then, ethical clearance was obtained from APHI (Ref No.:‐ አ.υ.ጤ.አ.ጤ.ቁ.ዳ ወ13‐02/11), and official permission was received from Bahir Dar City Administration Health Bureau. Butcheries’ owner permission was asked to take a raw meat sample, and the interviewee gave verbal consent by explaining the objectives of the study; their right and their participation are fully voluntary. To ensure confidentiality, any personal or butchery‐identifying information was not collected, and it was managed with a unique code.

## Conflicts of Interest

The authors declare no conflicts of interest.

## Data Availability

All the required information is included in the manuscript. However, the raw datasets used and/or analyzed during the current study are available from the corresponding author on reasonable request.
